# Rare adrenal incidentaloma: ganglioneuroma

**DOI:** 10.1093/jscr/rjae352

**Published:** 2024-05-31

**Authors:** Yassine Daghdagh, Reda Safwate, Abderrahmane Doumer, Amine Moataz, Mohamed Dakir, Adil Debbagh, Rachid Aboutaieb, Abd Mellouki, Samira Benayad, Mehdi Karkouri

**Affiliations:** Department of Urology, CHU Ibn Rochd, 20000 Casablanca, Morocco; Department of Urology, CHU Ibn Rochd, 20000 Casablanca, Morocco; Department of Urology, CHU Ibn Rochd, 20000 Casablanca, Morocco; Department of Urology, CHU Ibn Rochd, 20000 Casablanca, Morocco; Department of Urology, CHU Ibn Rochd, 20000 Casablanca, Morocco; Department of Urology, CHU Ibn Rochd, 20000 Casablanca, Morocco; Department of Urology, CHU Ibn Rochd, 20000 Casablanca, Morocco; Department of Anatomopathology CHU Ibn Rochd, 20000 Casablanca, Morocco; Department of Anatomopathology CHU Ibn Rochd, 20000 Casablanca, Morocco; Department of Anatomopathology CHU Ibn Rochd, 20000 Casablanca, Morocco

**Keywords:** urology, ganglioneuroma, retroperitoneal tumor, oncology

## Abstract

Ganglioneuroma (GN) is a rare, benign neurogenic tumor that develops from sympathetic ganglion cells. It occurs mainly in the retroperitoneal region. Adrenal localization is rare. We report a case of adrenal ganglioneuroma in a 22-year-old woman with no previous history of the disease. The tumor was discovered incidentally on an entero scan ordered as part of the etiological assessment for chronic diarrhea. The diagnosis was confirmed by pathological examination.

## Introduction

Ganglioneuroma (GN) is a benign, well-differentiated nerve tumor of children and young adults, consisting of mature sympathetic ganglion cells and nerve fibers, located in the adrenal gland (20%), along the sympathetic chain and particularly in the posterior mediastinum (40%) and retroperitoneum (30%) [1].

It belongs to the group of neurogenic tumors, developing at the expense of the sympathetic ganglion chains, a group that also includes ganglio-neuroblastomas and neuroblastomas [2].

These tumors can present a problem in terms of clinical and radiological diagnosis. Diagnosis is confirmed by pathological examination of the adrenalectomy specimen.

We report a case of adrenal GN recently observed in our practice.

## Case report

The patient was 22 years old with no previous pathological history. As part of the etiological work-up for anemia with chronic diarrhea, an enteroscan and colonoscopy were indicated.

**Figure 1:**
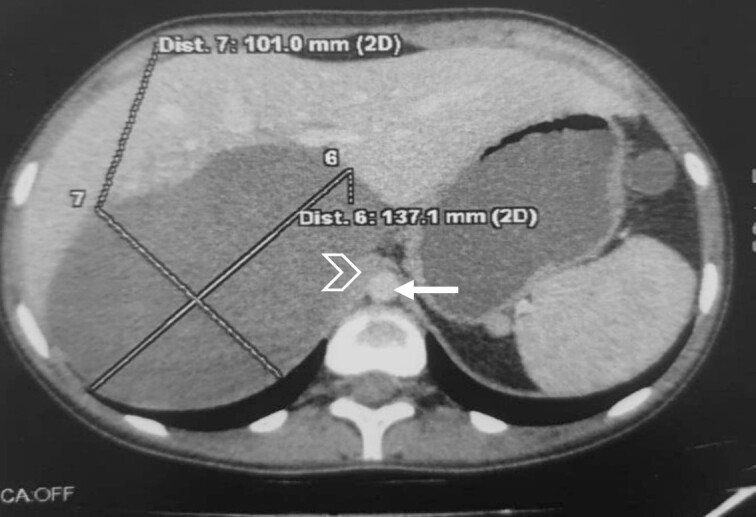
Large right retroperitoneal mass, hypodense, oval, well defined, with regular contours (arrow: aorta; arrowhead: IVC).

The CT scan incidentally revealed an adrenal incidentaloma in the form of a large right retroperitoneal mass measuring 137 × 101 × 114 mm^3^, occupying the adrenal cavity, oval, well defined, with regular contours, hypodense, containing areas of fluid density and others of tissue density without significant contrast enhancement, with a wall showing focal thickening of tissue density with low contrast uptake (Fig. 1).

The CT scan also revealed stenosing ileal parietal thickening suggestive of Crohn’s disease, which was confirmed by colonoscopy with biopsy.

**Figure 2:**
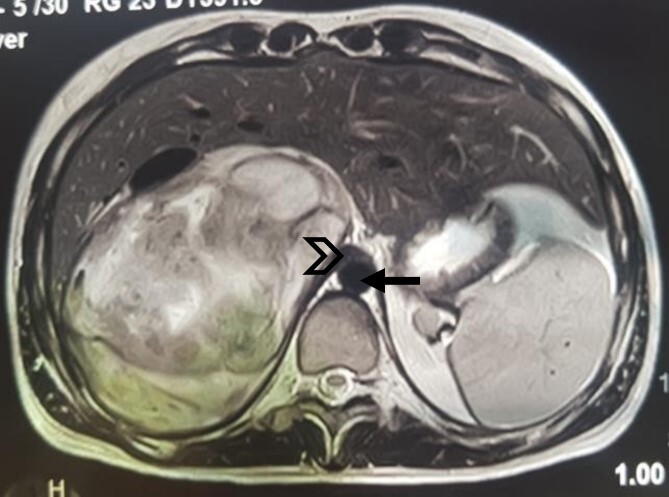
MRI, well-limited oval right adrenal mass, slightly heterogeneous mixed T2 signal (arrow: aorta; arrowhead: compressed IVC).

**Figure 3:**
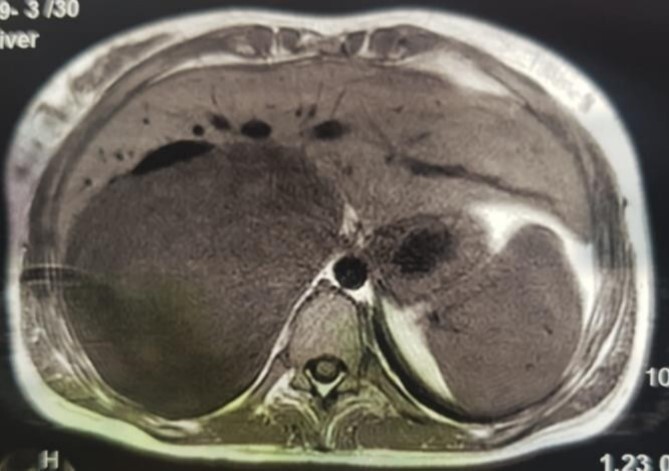
MRI, voluminous lesion of the right adrenal medulla in hyposignal T1.

An MRI angiogram to assess the vascular relationships of the tumor revealed a 125 × 110 × 95 mm^3^ well-bounded, oval, slightly heterogeneous right adrenal mass with a mixed T2 signal and hypointense T1 signal. Angiographic sequences showed no portal venous thrombosis; the lesion was adherent to the right liver and the inferior vena cava (IVC), which was compressed but remained permeable, and came into contact with the superior mesenteric artery and the superior mesenteric vein, which were also permeable. The hepatic pedicle was also slightly pushed forward (Figs. 2 and 3).

Blood glucose, renal and liver functions were normal. Plasma cortisol, urinary free cortisol and urinary methoxylates were also normal. Given the appearance of a non-secreting adrenal incidentaloma, the patient underwent lumbotomy first, converted into thoraco-phreno-lumbotomy due to complexity of the mass and adhesions to the IVC and the liver.

**Figure 4:**
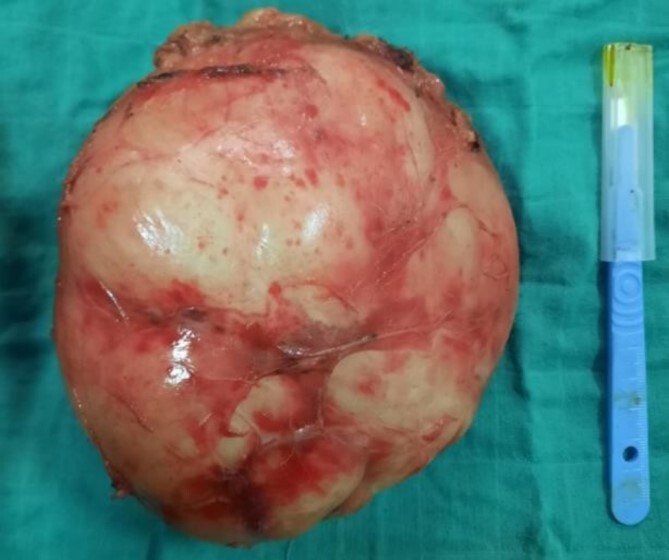
Adrenal piece, 14 × 13 × 9 cm^3^, 860 g, smooth, encapsulated surface

A right adrenalectomy was performed (Fig. 4). The mass was adherent to the liver, and its release was progressive, with meticulous dissection, especially in contact with the large vessels. Blood loss was not significant.

On macroscopic examination, the adrenal specimen measured 14 × 13 × 9 cm^3^ and weighed 860 g. The surface was smooth and encapsulated.

On section, it was whitish in colour, fasciculated and poly-lobed, with a firm consistency and myxoid changes. The adrenal parenchyma bulged out at the periphery and the surface measured 5 × 1.5 × 0.3 cm^3^.

**Figure 5:**
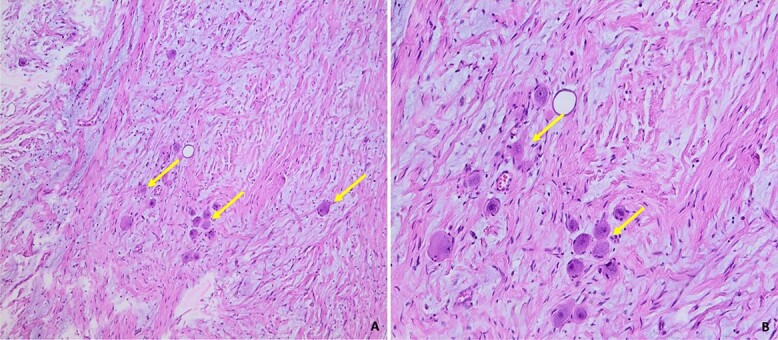
Histological images of our patient’s GN, showing a fusocellular proliferation of Schwanian-type cells with the presence of mature ganglion cells, isolated or grouped in piles (arrow). The stroma is fibrous and myxoid (Photo A: under standard coloration, enlargement x100) (Photo B: under standard coloration, enlargement x200).

Histological examination showed that the neoplasm was well circumscribed and delimited by a fibrous capsule. It described a predominantly fusocellular background, with moderate cellularity, diminished in places and dissociated by myxoid changes (Fig. 5).

The cells were spindle-shaped of the Schwanian type, undulating with elongated nuclei with fine chromatin, with no detectable cyto-nuclear atypia or atypical mitosis.

This Schwanian background was interspersed with large, heterogeneously dispersed mature ganglion cells. These cells had granular eosinophilic cytoplasm and large rounded nuclei with prominent eosinophilic nucleoli. There was no evidence of necrosis. The adrenal parenchyma, which was compressed at the periphery, was found preserved and regular in appearance.

Given these features, the diagnosis of adrenal GN was accepted. The post-operative course was normal and the patient presented no particular complications. The drain was removed after 4 days, and the patient was discharged after 5 days.

## Discussion

GN is a rare benign tumor of neuroectodermal origin. Like neuroblastoma and ganglioneuroblastoma, this tumor develops from the sympathetic nervous system.

GN develops along the sympathetic chain that runs from the neck to the pelvis. Retroperitoneal lymph nodes are the most common after mediastinal lymph nodes. The neck, anterior mediastinum and gastrointestinal tract are also possible sites, but are more rare [3].

It occurs at all ages, but is most common in children and young adults. Females are more often affected, with a sex ratio of 0.75 [4, 5].

Clinically, GN is often asymptomatic, discovered incidentally during a radiological work-up for another condition, as in our case, the tumor was discovered during investigation of chronic diarrhea. Occasionally, it may manifest as non-specific abdominal pain, an abdominal mass or it may be discovered during investigations for urinary, neurological, vascular or digestive signs caused by compression of neighboring organs.

Generally, GNs are non-functional, with a normal hormone profile [6]. In most cases, hormone secretion is normal. GN is generally a non-secreting tumor, but some authors report rare cases of GN with secretion of catecholamines or vasoactive intestinal polypeptide, responsible for diarrhea and arterial hypertension [7, 8].

Radiological diagnosis of these tumors is difficult. However, imaging enables the location of the tumor to be determined, as well as its relationship with neighboring organs, particularly blood vessels.

CT scans (without and with injection) are the first-line examination for adrenal glands. It confirms the retroperitoneal location and relationship of the tumor and predicts its resectability.

Some aspects are suggestive of GN: a well-limited ovoid tumor, with regular contours and very homogeneous contents, poorly vascularized, not invading neighboring vascular structures, located along the sympathetic chains, asymptomatic in a young or middle-aged subject [9].

Magnetic resonance imaging is also effective in differentiating between benign and malignant masses. It shows a tumor with a homogeneous hypo-signal in T1 and a hyper or iso-signal in T2, depending on the amount of stroma contained in the lesion. Contrast enhancement after injection of Gadolinium is not specific [10, 11].

Histological analysis of the surgical specimens provides a definitive diagnosis, allowing a complete analysis to rule out a neuroblastomatous contingent and also a pheochromocytoma within the GN. Macroscopically, GN is well limited, lobulated and sometimes encapsulated. The tumor is variable in size and often bulky. On cross-section, it is smooth, firm, gray-white and fasciculated. The presence of necrotic or haemorrhagic changes should raise suspicion of an immature contingent. Histologically, GN consists of mature lymph node cells with abundant eosinophilic cytoplasm and eccentric nuclei with a prominent nucleolus. The background is of variable abundance, made up of Schwann cells, sometimes organised in bundles. [9].

The differential diagnosis is essentially with ganglioneuroblastoma and neuroblastoma, but these tumors are suspected if there are radiological signs of locoregional invasion and if the tumor is infiltrative intraoperatively. GN is composed of mature ganglion cells and a stroma containing nerve cells and a Schwanian contingent, whereas neuroblastoma and ganglioneuroblastoma are composed of more immature ganglion cells with a greater potential for progression [12].

The main aspect of treatment is surgical removal, which is more difficult when the tumor is large and has close links with neighboring structures, particularly the large vessels (IVC and aorta). Treatment should be carried out early to confirm the nature of the mass and prevent it increasing in volume and compressing adjacent structures.

GN develops slowly. Their prognosis is good in the event of complete removal [10]. Local recurrence is possible. Malignant transformation into a ganglioneuroblastoma has been reported but remains rare, hence prolonged surveillance is important [13].

## Conclusion

Retroperitoneal GNs are rare benign tumors. They generally develop quietly, which explains why they are only discovered at the stage of a large tumor.

Despite advances in imaging, they continue to pose both diagnostic and therapeutic problems, particularly because of their relationship with neighboring organs, which makes them difficult to resect.

The possibility of local recurrence requires regular monitoring.

## Conflict of interest statement

None declared.

The examination of the patient was conducted in accordance with the Declaration of Helsinki Principles. Written informed consent was obtained from the patient for publication of this article.
